# Adsorption activity of coconut (*Cocos nucifera* L.) cake dietary fibers: effect of acidic treatment, cellulase hydrolysis, particle size and pH

**DOI:** 10.1039/c7ra13332d

**Published:** 2018-01-12

**Authors:** Yajun Zheng, Yan Li, Jianguo Xu, Gang Gao, Fuge Niu

**Affiliations:** Institute of Food Sciences of Shanxi Normal University Linfen 041004 China zyj_coconut@163.com +86-0357-2051482 +86-13976563642; College of Life Sciences and Food Engineering of Hebei Engineering University Handan 056038 China; The School of Food Science and Biotechnology, Zhejiang Gongshang University Hangzhou 310018 China

## Abstract

The effects of acidic treatment, cellulase hydrolysis, particle size distribution and pH on the adsorption capacity of defatted coconut cake dietary fibers (DCCDF) were studied. The results demonstrated that cellulase hydrolysis could significantly improve the soluble dietary fiber content, water holding ability and adsorption ability of DCCDF on cholesterol, bile and nitrite ions. Acidic treatment enhanced the oil holding capacity and adsorption ability in cholesterol and nitrite ions. Moreover, the adsorption ability of DFs in cholesterol, nitrite and bile all increased with reduced particle size (250 to 167 μm), and DCCDF demonstrated a higher adsorption capacity at pH 2.0 than at pH 7.0. The change in adsorption capacity of DCCDF might be suitable for application in the food industry as a low-calorie and cholesterol lowering functional ingredient.

## Abstract 2

This paper provides valuable information on the effects of cellulase hydrolysis, acidic treatment, particle size distribution and pH on the adsorption activity of coconut cake dietary fibers.

## Graphical abstract

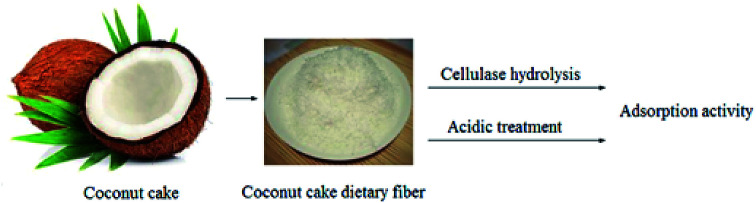

## 1. Introduction

Recently, dietary fibers (DF) derived from cereal, fruit and vegetable by-products have received more and more attention for their low cost and beneficial effects on human health, including reduced risk of coronary heart disease, diabetes and some forms of cancer.^1^ In food and non-food industries, the usage of DFs is dependent on their physicochemical and functional properties, which can be modified by extraction methods, processing conditions and particle size.^2^ Adsorption activity is one of the most important functional properties of DFs. It was evident that DFs with high binding ability could effectively accelerate the excretion of bile acids, in turn reducing the adsorption of bile in gastrointestinal digestion and preventing epithelial cell and DNA damage.^3^ Some DFs with high adsorption activity of oil could reduce plasma cholesterol levels as well as the incidence of atherosclerosis.^4^ Some DFs have even demonstrated high adsorption ability of hazardous substances such as heavy metals, nitrite and others.^1^ Moreover, previous study also found that adsorption activity of DFs was correlated with monosaccharide composition, soluble dietary fiber (SDF) content, water holding capacity (WHC), water swelling capacity (WSC) and oil holding capacity (OHC), and contributed to the glucose dialysis retention index (GDRI) and emulsifying property of DFs.^5^

Most of the DFs derived from cereal, fruit and vegetable by-product could be classified as insoluble dietary fiber (IDF). IDF is composed of cellulose, hemicelluloses and lignin, which contains several functional groups such as alcohols, aldehydes, ketones, carboxylic acid, phenolic and ether linkages.^6^ These groups have a strong affinity to bind water, oil or toxic metal ions, therefore positively related with the adsorption ability of DFs. However, typical processing methods of DFs like alkali extraction and enzymatic hydrolysis can't cause denaturation of linkages among polysaccharides, hence can't adequately expose these groups or binding sites. Thus, several physical, chemical and enzymatic treatments such as micronization, shear emulsifying, acidic treatment, as well as cellulase and/or xylanase hydrolysis have been reported.^3,4,6^ It was evident that cellulase hydrolysis could increase SDF content of DFs, and improved some physicochemical and functional properties of DFs such as WHC, emulsifying properties, entrapment capacity and adsorption ability.^4,7^ Jing *et al.*^6^ found that acidic treatment could improve the porosity and crystallinity, in turn enhance oil hold capacity of rice bran dietary fiber. Other than that, changes in particle size and pH may influence the structure, porosity and surface structure of DFs, resulting in modifications of physicochemical properties.^1,8^ It was reported that reduction in particle size could improve the WHC, SWC and OHC of cumin DFs, and DFs could exhibited higher adsorption capacity at low pH values.^2,3,7^

Coconut cake, a byproduct of the coconut oil industry, is a good source of dietary fibre, for it has a high content of DF (14%) and a low cost.^9^ However, coconut cake dietary fiber is also classified as IDF since its SDF content is only 4%.^10^ Although some physicochemical properties of coconut dietary fibre from virgin coconut oil residue and coconut milk residue have been studied,^10^ there is little information on the modification of physicochemical properties especially the adsorption ability of coconut cake dietary fibre caused by different treatment.

In this study, the objectives of this paper are (1) to evaluate the effects of acidic treatment and cellulase hydrolysis on the adsorption activity of DCCDF and (2) to study the modifications of adsorption activity induced by changes in pH and particle size.

## 2. Materials and methods

### 2.1 Materials

Coconut (variety: China Tall coconut, 12 months maturity) cake was obtained from the Coconut Grand View Garden, Wenchang, Hainan province, China. Papain (1.0 × 10^4^ U), α-amylase (2.0 × 10^4^ U), glucoamylase (5.0 × 10^4^ U) and cellulase (3.0 × 10^5^ U) were purchased from Shanghai DINGUO Biotech. Co., Ltd. (China). The monosaccharide standards including l-arabinose, d-galactose, aminoglucose, and others were purchased from Sigma Co., USA. Cholesterol, sodium nitrite, sodium acetate and other chemicals and reagents were of analytical grade.

### 2.2 Defatted coconut cake (DCC) preparation

One kilogram of coconut cake was dried at 50 °C for 4 h in a dryer, grated into a crude powder with a mill and then defatted three times with *n*-hexane (1 : 10, g mL^−1^). The defatted powder was milled again and passed through a sieve of 40 meshes to get a fine powder of DCC.

### 2.3 DF extraction, acidic treatment and enzymatic hydrolysis

#### 2.3.1 Preparation of DCCDF

Following the method of Ma & Mu,^3^ 100 g of DDC was suspended in 1000 mL phosphate buffer (0.1 M), 1 g of α-amylase was added and the solution was stirred gently at 90 °C for 2 h. The mixture was cooled and adjusted to pH 7.0, and 0.5 g papain was added. After incubation at 50 °C for 2 h, the reaction solution was adjusted to pH 2.0, mixed with 0.5 g glucoamylase and incubated at 60 °C for 2 h. The mixture was incubated at 100 °C for 10 min to inactivate the enzymes, and cooled to room temperature (RT) and filtered using 100-meshe linen. The residue was collected, washed with deionized water, dried at 50 °C for 12 h, and then DCCDF was obtained.

#### 2.3.2 Acidic treatment

Fifty grams of DCCDF was suspended in 500 mL of 1 M NaOH. After stirring at 60 °C for 2 h, the suspension was filtered through linen, and the residue was collected. This residue was continuous soaked in 1 M HCl at 60 °C for 30 min, then neutralized, filtered, washed with deionized water and dried in a forced air-oven at 60 °C for 8 h.^6^ Then DCCDF with acidic treatment (DCCDF-A) was obtained.

#### 2.3.3 Enzymatic hydrolysis

Fifty grams of DCCDF described above were suspended in 500 mL of phosphate buffer (0.1 M, pH5.0) and mixed with 0.3 g cellulase. After incubation at 50 °C for 1 h, the mixture was heated at 100 °C for 10 min and then cooled to RT and filtered using 100-meshe linen.^4^ The residue was collected, dried at 50 °C for 12 h and named as DCCDF-C.

### 2.4 Chemical and monosaccharide composition analysis

Moisture (method 925.09), protein (method 955.04), fat (method 920.39), starch (method 996.11), ash (method 942.05), total DF, IDF, and SDF (method 991.43) contents were measured by the AACC official methods (1986, 1995).^11,12^ The polyphenol content was determined by the Folin–Ciocalteu colorimetric method.^13^ The monosaccharide composition of DCC and DFs was measured by a modified method reported by Chau and Huang.^14^ Briefly, DFs were hydrolyzed in 12 M H_2_SO_4_ at 40 °C for 1 h. The hydrolysate was then diluted with deionized water to make the final concentration of H_2_SO_4_ to 1 M and autoclaved at 121 °C for 1 h. The obtained hydrolysate was neutralized with KOH, filtered through a 0.45 μm membrane and then injected into an ICS-5000 HPLC system (DIONEX Co., Sunnyvale, USA) which was equipped with a pulse ampere detector. The mobile phase was H_2_O/NaOH/NaAc: 78.2/1.8/20 at a flow rate 0.5 mL min^−1^, at 25 °C.

### 2.5 Particle size distribution

The obtained DCC and DCCDF, DCCDF-A and DCCDF-C were ground into a fine powder and separated by a sieve shaker (Model VE 100, Retch, Germany) with a series of sieves (mesh No. 40, 60, 80 and 100). Each sample was placed in the top sieve with the largest mesh and shaken for 5 min at amplitude setting of 2 mm, disassembled and stirred lightly, then shaken for additional 5 min. The particle size distribution parameters expressed as Sautermean diameter *D*_3,2_ (μm) and specific surface area of the DFs obtained from the sieving mesh were determined by a Laser Diffraction Particle Size Analyzer (MS3000, Malvern instruments Ltd., UK).

### 2.6 Scanning electron microscopy (SEM)

The surface and microstructure of DFs were observed by SEM (S-3400 scanning electron microscope, Hitachi, Ltd., Tokyo, Japan) at 20 kV. Powder samples were mounted on metal stubs and sputter-coated with a 10 nm gold and palladium layer by Ion Sputter (Bio-Rad SC-500).

### 2.7 Physicochemical properties

#### 2.7.1 Water holding capacity (WHC)

WHC was determined following the method of Sangnark and Noomhormb.^15^ Samples (1 g) were mixed with 30 mL of distilled water at room temperature for 2 h. The excess water was removed by allowing the wet sample to drain on a fine-meshed wire screen. A portion of the wet sample on the screen was carefully removed, weighed (as wet weight) and dried to constant weight (dry weight) in a forced-air oven (110 °C). WHC was defined as follows:

1 WHC (g g^−1^) = (wet weight − dry weight)/dry weitht

#### 2.7.2 Oil adsorption capacity (OAC)

OAC was determined by the method described by Ma & Mu.^3^ DFs (1 g) was mixed with soybean oil in a centrifugal tube and left for 1 h at RT. The mixture was then centrifuged at 1500 × *g* for 10 min the supernatant decanted and the pellet recovered by filtration through a linen mesh. OAC was expressed as follows:

2 OAC (g g^−1^) = (pellet weight − dry weight)/dry weight

### 2.8 Adsorption capacity

#### 2.8.1 Cholesterol binding ability (CBA)

Aliquots of samples (2 g) with different particle sizes were mixed with 50 mL cholesterol solution (0.1 mg mL^−1^, pH 2.0) and incubated in a shaking water bath at 37 °C for 150 min. At 30 min intervals, the suspension was centrifuged at 3000 × *g* for 10 min. Then 0.1 mL of the mixture was collected and the concentration of cholesterol was determined using the *O*-phthalaldehyde sulfuric acid colorimetric method (OPA).^16^ CBA was calculated as follows:

3 CBA (%) = (1 − *C*_*t*_/*C*_0_) × 100

where *C*_*t*_ was the cholesterol concentration of the reaction solution at the tested time, and C_0_ was the original cholesterol concentration (0.1 mg mL^−1^).

The effect of different pH values on CBA was also examined by comparing the CBA of samples at pH 2.0 and pH 7.0.

#### 2.8.2 Bile salt binding ability (BSBA)

Following the modified method of Peerajit *et al.*^5^ with some modifications, 2 g of samples with different particle sizes was mixed with 100 mL of sodium cholate (1 mg mL^−1^, pH 2.0) and incubated in a shaking water bath at 37 °C for 5 h. At 1 h intervals, the suspension was centrifuged at 3000 × *g* for 10 min, and then 1 mL of mixture was collected and mixed with 6 mL of 45% sulfuric acid and 1 mL of 0.3% furaldehyde. After incubation at 65 °C for 30 min, the absorbance at 620 nm was measured. BSBA was calculated as follows:

4 BSBA (%) =(1 − *C*_*t*_/*C*_0_) × 100

where *C*_*t*_ was the sodium cholate concentration of the reaction solution at the tested time, and *C*_0_ was the original sodium cholate concentration (1 mg mL^−1^).

The effect of different pH values on BSBA was also researched by comparing the BSBA of samples at pH 2.0 and pH 7.0.

#### 2.8.3 Adsorption activity of nitrite ions (AAN)

Two grams of samples with different particle size were mixed with 100 mL of sodium nitrite (0.1 mM, pH 2.0) and incubated in a shaking water bath at 37 °C for 150 min. At 30 min intervals, 2 mL of the mixture was collected and mixed with 1.125 mL of ammonium chloride buffer, 0.625 mL of 6% HAC, 0.625 mL of *p*-aminobenzene sulfonic acid and 0.625 mL of *N*-1-naphthylethylenediamine dihydrochloride.^17^ After incubation in the dark for 25 min, the absorbance at 550 nm was measured. AAN was calculated as follows:

5 AAN (%) = (1 − *A*_*t*_/*A*_0_) × 100

where *A*_*t*_ was the absorbance at tested time, and *A*_0_ was the original absorbance at 550 nm.

In addition, the effect pH on AAN was studied by comparing the AAN of samples at pH 2.0 and pH 7.0.

#### 2.8.4 Statistical analysis

All of the results were the mean values of triplicates. The data were subjected to analysis of variance and Duncan's test with a confidence interval of 95% calculated to compare mean values.

## 3. Results and discussion

### 3.1 Proximate composition

The proximate composition of DCC and the DFs (DCCDF, DCCDF-A and DCCDF-C) is shown in Table 1. Compared to DCC, the TDF content of the three DFs was higher while the fat and protein content was lower, which was in accordance with the report of Yalegama *et al.*^10^ DCCDF-A produced by acidic treatment, exhibited a lower SDF content (2.16 ± 0.51 g/100 g) than that of DCCDF (*P* < 0.05), indicating that the acidic treatment led to reduction in SDF. In contrast, the SDF content of DCCDF-C was much higher than that of DCCDF (*P* < 0.05), suggesting that cellulase could cause the degradation of hemicelluloses and cellulose, resulting in increase in SDF content. Furthermore, the TDF content of DCCDF and DCCDF-C was higher than that of DFs from rice bran (27.04 g/100 g), sesame coat (42.00 g/100 g) and deoiled cumin (46.01 g/100 g),^1,3,6^ indicating that they could be used as functional ingredient.^18^

**Table 1:** Effects of acidic treatment and cellulase hydrolysis on the proximate composition and monosaccharide composition of coconut cake dietary fiber^a^

Proximate composition	DDC	DDCDF	DDCDF-A	DDCDF-C
Moisture (g/100 g)	5.75 ± 0.16 a	2.95 ± 0.43 d	3.95 ± 0.14 c	4.12 ± 0.21 b
Fat (g/100 g)	3.22 ± 0.22b	1.96 ± 0.20 b	0.65 ± 0.03 b	0.42 ± 0.08 b
Protein (g/100 g)	19.95 ± 0.56 a	5.25 ± 0.19 c	2.10 ± 0.12 d	4.66 ± 0.32 c
Soluble carbohydrate (g/100 g)	10.59 ± 0.32 c	24.18 ± 1.55 b	3.74 ± 0.12 d	32.01 ± 2.04 a
Ash (g/100 g)	2.02 ± 0.15 a	2.14 ± 0.20 a	0.22 ± 0.01 c	2.32 ± 0.16 a
TDF (g/100 g)	52.31 ± 4.02 c	84.49 ± 3.37 a	70.00 ± 1.14 b	86.05 ± 3.25 a
SDF (g/100 g)	7.83 ± 0.58 c	19.33 ± 1.06 b	2.16 ± 0.51 d	29.95 ± 1.12 a
IDF (g/100 g)	44.48 ± 2.22 c	65.16 ± 3.64 a	67.34 ± 4.16 a	56.16 ± 3.64 b
IDF/SDF	5.68 ± 0.32 c	3.37 ± 0.11 d	7.08 ± 0.24 b	1.87 ± 0.24 e
Polyphone (mg/100 g)	5.43 ± 0.22 c	2.39 ± 0.18 d	2.01 ± 0.12 d	7.62 ± 0.31 b
Arabinose (g kg^−1^)	13.13 ± 0.25 a	13.19 ± 0.14a	ND	ND
d-Galactose (g kg^−1^)	16.85 ± 0.26 b	16.90 ± 0.42 b	27.15 ± 1.14 a	28.23 ± 2.18 a
Glucose (g kg^−1^)	19.13 ± 1.43 b	19.12 ± 1.58 b	34.67 ± 3.01 a	32.24 ± 1.45 a
Xylose (g kg^−1^)	23.20 ± 1.25 c	23.13 ± 1.51 c	37.55 ± 1.69 b	39.53 ± 2.84 a
Fructose (g kg^−1^)	27.69 ± 3.44a	27.66 ± 2.13 a	ND	ND

a DCC, defatted coconut cake; DCCDF, defatted coconut cake dietary fiber; DCCDF-A, defatted coconut cake dietary fiber treated by acid; DCCDF-C, defatted coconut cake dietary fiber with cellulase hydrolysis; TDF, the total dietary fiber; SDF, soluble dietary fiber; IDF, insoluble dietary fiber. Different small letters (a–e) in the same line meant significant difference (*P* < 0.05). “ND” meant undetectable.

The monosaccharide composition of the DFs is also shown in Table 1. Five types of monosaccharide were detected in DCC, of which arabinose and fructose was the major. All DCCDF, DCCDF-A and DCCDF-C exhibited higher glucose content but lower arabinose and fructose content than DCC (*P* < 0.05). The reason may be that the pectic substances and hemicelluloses of DCC such as xyloglucan were significantly lessened by the acidic treatment and cellulase hydrolysis, leading to an increase in sugar content.^3^ Similar result was obtained by other researchers.^4,6^

### 3.2 Particle size distribution

The particle sizes distribution and specific surface area of DFs were shown in Table 2. Obviously, the particle size of the DFs decreased with increasing sieve mesh size, while the specific area increased as the sieve mesh size increased. The DFs with particle sizes less than 100 μm compose 19.60%, 32.24% and 56.01% of DCCDF-A, DCCDF and DCCDF-C respectively, which were all higher than that of DCC (19.60%). Similar trend was obtained on deoiled cumin and seaweed dietary fibers.^3,19^ Within same mesh size, DCCDF-C showed the smallest particle size and biggest specific surface (171.06 m^2^ kg^−1^) among the DFs, possibly attributed to the degradation of cellulose or hemicellulose caused by cellulase.^7^

**Table 2:** Particle size distributions, water holding capacity (WHC) and oil holding capacity (OHC) of dietary fibers obtained from defatted coconut cake by different treatment^a^

DFs	Sieving mesh sizes	*D*_3,2_ (μm)	Surface area (m^2^ kg^−1^)	Distribution (%)	WHC (g g^−1^)	OHC (g g^−1^)
DCCDF	Unscreened (>250 μm)	235.0 b	25.51 gh	8.82 ± 1.30 lm	13.08 ± 0.36 b	9.91 ± 1.74 b
	40 (250–167 μm)	184.2 c	30.54 g	26.19 ± 1.58 ef	12.75 ± 0.20 b	6.34 ± 0.20 ef
	60 (167–125 μm)	148.0 d	40.59 f	32.75 ± 0.07 bc	11.59 ± 0.56 c	6.14 ± 0.16 ef
	80 (125–100 μm)	60.6 f	99.02 c	20.89 ± 1.56 h	10.64 ± 0.06 d	6.53 ± 0.14 ef
	100 (<100 μm)	46.2 g	121.99 b	11.35 ± 1.64 jk	9.90 ± 0.52 ef	6.53 ± 0.34 ef
DCCDF-A	Unscreened (>250 μm)	224.7 b	28.19 g	7.07 ± 0.95 mn	3.66 ± 0.15 k	11.81 ± 0.37 a
	40 (250–167 μm)	201.4 bc	31.42 g	31.09 ± 0.48 cd	2.24 ± 0.11 l	9.69 ± 0.53 b
	60 (167–125 μm)	179.0 c	33.51 g	35.89 ± 0.56 a	4.19 ± 0.08 jk	7.95 ± 0.39 c
	80 (125–100 μm)	92.4 e	53.88 ef	15.07 ± 0.43 i	4.55 ± 0.02 j	6.97 ± 0.26 de
	100 (<100 μm)	69.3 f	88.74 d	10.88 ± 1.47 kl	3.77 ± 0.03 k	6.09 ± 0.30 ef
DCCDF-C	Unscreened (>250 μm)	233.0 b	29.97 g	9.21 ± 1.13 l	8.39 ± 0.55 h	6.69 ± 0.65 de
	40 (250–167 μm)	153.7 d	48.26 f	34.29 ± 0.53 ab	11.25 ± 0.03 c	4.42 ± 0.14 h
	60 (167–125 μm)	91.5 ef	63.17 e	24.84 ± 1.81 fg	13.87 ± 0.22 a	4.26 ± 0.27 h
	80 (125–100 μm)	49.9 g	123.65 b	19.17 ± 1.17 h	10.42 ± 0.14 de	4.54 ± 0.84 h
	100 (<100 μm)	39.7 h	171.06 a	12.00 ± 1.62 jk	8.92 ± 0.21 g	4.59 ± 0.68 h

a DCC, defatted coconut cake; DCCDF, defatted coconut cake dietary fiber; DCCDF-A, defatted coconut cake dietary fiber treated by acid; DCCDF-C, defatted coconut cake dietary fiber with cellulase hydrolysis; different small letters (a–n) in the same column meant significant difference (*P* < 0.05).

### 3.3 SEM

**Fig. 1:**
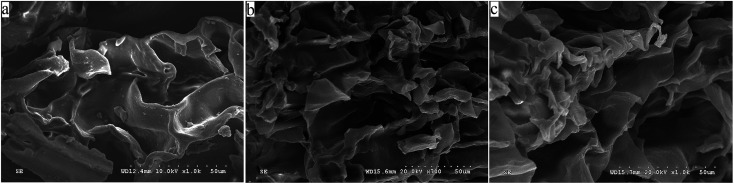
Scanning electron microscopy (SEM) of defatted coconut cake dietary fiber (DCCDF), defatted coconut cake dietary fiber treated by acid (DCCDF-A), and defatted coconut cake dietary fiber with cellulase hydrolysis (DCCDF-C).

SEM images of DCC and the DFs are shown in Fig. 1. Compared to DCC and DCCDF, both DCCDF-A and DCCDF-C exhibited a characteristic honeycomb structure with a greater number of holes and cracks (Fig. 1a–c), possibly because that the acidic treatment and enzymatic hydrolysis removed proteins or starch around the DF bundles,^6^ which was also contributed to the changes in surface area of the DFs (Table 2). Similar result was obtained by other researchers.^3,19,20^

### 3.4 Water holding capacity

As shown in Table 2, the highest WHC (13.87 ± 0.22 g g^−1^) was found on DCCDF-C with particle size of 91.5 μm, perhaps due to the high content of SDF (Table 1) and the honeycomb structure (Fig. 1c). The SDF content was positively correlated with WHC of DFs, and cellulase hydrolysis could break the intermolecular hydrogen bonds of cellulose and hemicelluloses, improve the exposure of hydrated hydroxide, carboxyl groups and capillary action of fiber, in turn leading to increment in WHC.^7^ Although it was reported that dilute acid treatment could enhance WHC of fibers by removing starch, protein and hemicellulose in fiber, improving the specific surface area of fiber and exposing more hydrophilic groups,^5,6^ DCCDF-A produced with acidic treatment showed much lower WHC than that of DCCDF (*P* < 0.05), probably attributed to its low SDF content (Table 1). For the acidic treatment could cause denaturation of linkages among polysaccharides such as cellulose and hemicelluloses, leading to a loss of SDF which was positively correlated with WHC, finally decreased the WHC of DCCDF.^7^

Moreover, WHC of DCC and DCCDF decreased with decreasing particle size. In case of DCCDF-C, the WHC increased when the particle size decreased from 233 to 91.5 μm, but decreased when particle size further diminished. DFs may interact with water through the capillary structures, as a result of surface tension strength or/and by hydrogen bonds and dipole interactions.^3^ The increased specific surface area with decreasing particle size (Table 2) mainly contributed to the improvement of WHC, but the denaturation of linkages among polysaccharides in DFs during the milling can decrease the WHC.^3^ In addition, The WHC of DCC, DCCDF and DCCDF-C (12.74–13.87 g g^−1^) was much higher than that of DFs from sugarcane bagasse DFs (4.98–8.61 g g^−1^), maca DFs (8.39 g g^−1^) and deoiled cumin (3.38–6.96 g g^−1^),^3,15,20^ meaning that they can be used as a functional ingredient to avoid syneresis and modify the viscosity and texture of some formulated foods.^1^

### 3.5 Oil holding capacity

OHC of DFs is related to some functional properties such as the ability to prevent fat loss during food processing and the capacity to reduce serum cholesterol levels.^21^ DCCDF-A exhibited the highest OHC (11.81 ± 0.37 g g^−1^) among the samples, which was also higher than that of sugarcane bagasse DFs (3.26–7.34 g g^−1^) and cumin DFs (5.42–7.09 g g^−1^),^3,15^ possibly attributable to the surface properties (Fig. 1c) and the reduction of polar groups, uronic acid groups caused by the removal of starch and hemicelluloses (Table 1).^5,6^ In contrast, DCCDF-C demonstrated the lowest OHC (4.26–6.69 g g^−1^, Table 2), may be due to the increment of polar groups and hydrogen bonds caused by degradation of linkages among polysaccharide.^19^ Moreover, OHC of DFs decreased with decreasing particle size, possibly attributable to the increment of polar groups during milling.^3^ Similar trend was found on rice bran fiber.^6^

### 3.6 Adsorption capacity

#### 3.6.1 Bile salt binding ability

**Fig. 2:**
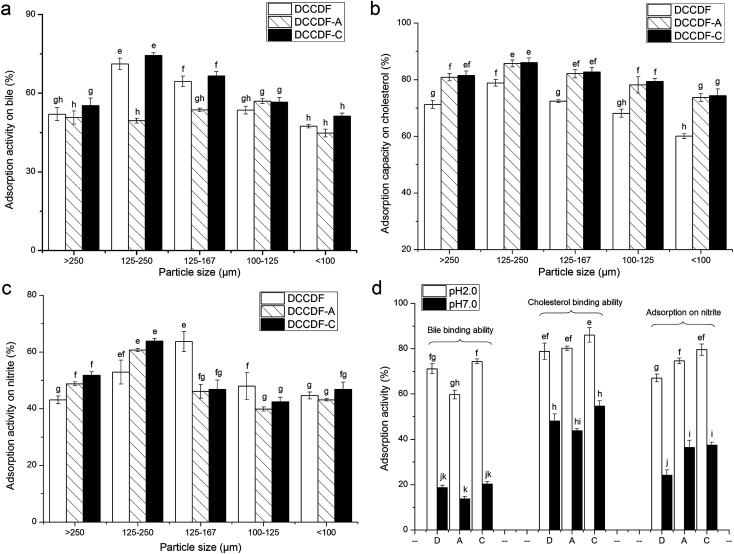
Effects of particle size (a–c) and pH (d) on the bile, cholesterol and nitrite ion adsorption capacity of defatted coconut cake dietary fiber (DCCDF), defatted coconut cake dietary fiber treated by acid (DCCDF-A), and defatted coconut cake dietary fiber treated by cellulase (DCCDF-C). (D) in *x*-axis of (d) DCCDF, (A) DCCDF-A, (C) DCCDF-C. Different small letters (e–k) on the bar means significant difference (*P* < 0.05).

DFs with high BSBA can effectively accelerate the excretion of bile acids, sequentially reducing adsorption in gastrointestinal digestion and preventing epithelial cell and DNA damage.^3^ As shown in Fig. 2a, DCCDF-C showed the highest BSBA (45.49–74.37%) among the samples, possibly due to the high content of SDF and WHC (Tables 1 and 2).^4^ In contrast, DCCDF-A showed lower BSBA than DCCDF, indicating the acidic treatment weaken BSBA of DCCDF. It was evident that adsorption activity of DFs was positively correlated with SDF and WHC.^3,5^ Moreover, the BSBA of the samples increased with decreasing particle size (250–167 μm), but reduced as the particle size further decreased. Reduction in particle size caused larger specific surface areas (Table 2), thereby lead to increment in BSBA of DFs. However, the decrease in particle sizes also meant more denaturation of hydrogen bonds and dipole forms, sequentially weakening the BSBA.^3^ In addition, all the DFs exhibited much higher BSBA at pH 2.0 than that at pH 7.0 (Fig. 2d). DFs can show more hydrophobic groups and higher ion exchange ability in acidic condition, leading to increase in adsorption capacity.^6^

#### 3.6.2 Cholesterol binding ability

Cholesterol serves as vital component for vertebrate cell membrane structure and function, and precise regulation of cholesterol homeostasis is essential.^22^ The cross-sectional studies demonstrated that enhanced cholesterol synthesis and reduced cholesterol absorption is a major risk factor for some diseases such as cardiovascular disease, type 2 diabetes and obesity.^23^ One of the most important beneficial effects of dietary fiber (whether SDF or IDF) is to reduce plasma cholesterol levels, as well as the incidence of atherosclerosis.^1,4^ As shown in Fig. 2b, DCCDF-C showed a higher CBA than DCCDF, indicating that the cellulase hydrolysis could improve the CBA of DCCDF, which was probably attributable to the higher SDF content and honeycomb structure (Fig. 1). Ma & Mu also found that DF sources containing more SDF could adsorb and bind more cholesterol and bile acid.^3^ Meanwhile, the acid treatment also enhanced the CBA of DCCDF, due to the higher OHC (Table 2).^4,6^ Moreover, result in Fig. 2d revealed that all the samples exhibited higher CBA at pH 2.0 than at pH 7.0, indicating that they could more effectively adsorb bile acid in the stomach than in the intestine.

#### 3.6.3 Adsorption activity on nitrite

Nitrate ions and nitrite ions are one of the world's major pollutants of drinking-water resources. Nitrites can be converted into carcinogenic nitrosamines which are harmful and toxic to the human body. It also can combine with hemoglobin in the blood and is the cause of “blue baby syndrome”.^17^ A high AAN indicates that DFs can effectively lower the adsorption of nitrite in gastrointestinal digestion and accelerate the excretion. As shown in Fig. 2c, both acid treatment and cellulase hydrolysis could improve the AAN of DCCDF. One reason was the higher content of SDF or the porous surface structure (Table 1 and Fig. 1b and c). It was evident that cellulase hydrolysis and acidic treatment could break the β-glycosidic linkages of cellulose and hemicelluloses, expose more carboxylic and hydroxyl phenolic groups, resulting in increment in AAN.^6^ Moreover, increase in AAN of DFs with deceasing particle size (250 to 167 μm) was also obtained, attributed to the increased surface area.^10^ Moreover, all samples also showed much higher AAN at pH 2.0 than that at pH 7.0 (Fig. 2d), corresponding to the BSBA. Previous studies also demonstrated that DFs derived from cereal, fruit and vegetable by-product exhibited higher adsorption ability in acidic conditions than in alkaline conditions.^5,17^

## 4. Conclusions

The results revealed that cellulase hydrolysis could improve the SDF content and WHC of DCCDF, in turn resulted in increase of adsorption ability in bile, cholesterol and nitrite ion. Acidic treatment could enhance the OHC, CBA and ANN of DCCDF, but reduced the BSBA. Moreover, reduction in particle size (from 250 to 167 μm) caused increases in BSBA, CBA and ANN. DFs exhibited higher adsorption activity at pH 2.0 rather than at pH 7.0. In general, DCC, DCCDF- A and DCCDF-C have potential applications in food and health products as promising low-calorie functional ingredients for fiber enrichment.

## Conflicts of interest

The authors declare that they have no competing interests.

## Supplementary Material
